# Unusual case of retroperitoneal hematoma and duodenal ulcerative bleeding after nephrectomy: Case report

**DOI:** 10.1097/MD.0000000000033765

**Published:** 2023-02-02

**Authors:** Yong Luo, Qing Li, Zhanchen Liao, Zhigang Luo

**Affiliations:** a Hengyang Medical School, University of South China; Trauma Centre & Emergency Department, The Second Affiliated Hospital of the University of South China, Hengyang, P.R. China; b Trauma Centre & Emergency Department, and Institute of Urology and Organ Transplantation, The Second Affiliated Hospital of the University of South China, Hengyang, P.R. China.

**Keywords:** gastrointestinal hemorrhage, gastrointestinal ulcer, radical nephrectomy, retroperitoneal hematoma, urinary tract infection

## Abstract

**Rationale:**

Retroperitoneal hematomas are relatively common in patients undergoing nephrectomy. Herein, we report an unusual case involving a giant retroperitoneal hematoma and subsequent duodenal ulcerative bleeding following a radical nephrectomy.

**Patient concerns:**

A 77-year-old woman was admitted to our hospital for lower back pain, and she had severe right hydronephrosis and a urinary tract infection.

**Diagnoses:**

The patient was diagnosed and confirmed as high-grade urothelial carcinoma.

**Interventions:**

After ineffective conservative treatments, a right radical nephrectomy and ureteral stump resection were performed. The patient received proton pump inhibitors to prevent stress ulcer formation and bleeding. On the first day post-surgery, she had normal gastrointestinal (GI) endoscopy findings. On the second day post-surgery, abdominal computed tomography revealed a retroperitoneal hematoma. Notably, 14 days post-surgery, massive GI bleeding occurred, and GI endoscopy identified an almost perforated ulcer in the bulbar and descending duodenum.

**Outcomes:**

The patient died on day 15 after surgery.

**Lessons:**

Duodenal ulceration and bleeding might occur following a retroperitoneal hematoma in patients treated with nephrectomy. Timely intervention may prevent duodenal ulcers and complications, and thus could be a promising life-saving intercession.

## 1. Introduction

Duodenal ulcers are the major cause of gastrointestinal (GI) bleeding, a potentially life-threatening clinical condition associated with high mortality in severe cases.^[1,2]^ Etiological studies have shown that bacterial infections, namely with *Helicobacter pylori*, are the most common cause for duodenal ulcers and account for as many as 95% of cases. Other common causative factors include regular long-term use of non-steroidal anti-inflammatory drugs (e.g. ibuprofen, naproxen sodium, ketoprofen). In addition, taking non-steroidal anti-inflammatory drugs in combination with other mediations (such as steroids, low-dose aspirin, selective serotonin reuptake inhibitors, anticoagulants) as well as stress can increase the risk of duodenal ulceration. It has also been noted that obstructive malignancies and external mechanical forces may result in intestinal ischemia and perforation. However, the exact mechanisms responsible for duodenal ulceration and perforation remain unclear. Until now, duodenal ulcers or bleeding after the occurrence of a retroperitoneal hematoma following a radical nephrectomy has not been reported.

In this case study, we report a 77-year-old woman who underwent a right radical nephrectomy and developed the procedure-related complication of a giant retroperitoneal hematoma with subsequent duodenal ulcerative bleeding. Sharing the experience and lesson acquired by conducting this case study is important given that the timely treatment of retroperitoneal hematomas should be strongly encouraged in patients with similar conditions after radical nephrectomy procedures. The early intervention of duodenal bleeding may be a promising life-saving intercession.

## 2. Case presentation

### 2.1. Chief complaints

A 77-year-old woman was admitted to our hospital complaining of lower back pain on the right side lasting longer than 6 months, and worsening pain with fatigue for 10 days.

### 2.2. History of present illness

One month prior to admission, the patient was diagnosed with a right renal abscess and urinary tract infection (UTI) and was treated with percutaneous nephrostomy and carbapenem antibiotics at a different hospital.

### 2.3. History of past illness

The patient had a medical history of hypertension, gastric disease, and right kidney stones.

### 2.4. Personal and family history

The patient has no known family history of related diseases.

### 2.5. Physical examination upon admission

Physical examination was performed upon admission, and no abdominal mass was detected in the patient.

### 2.6. Imaging examinations

**Figure 1.**
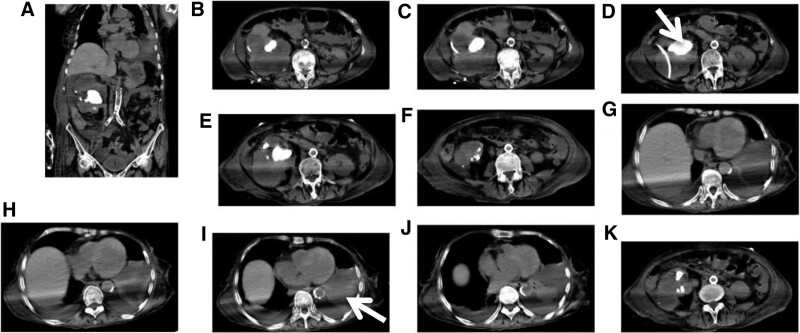
**Preoperative computed tomography (CT) images.** Preoperative CT imaging showed bilateral pleural effusion, bilateral interstitial pneumonia, multiple kidney stones, and severe right hydronephrosis in the patient. The arrow in the upper panel D indicated kidney stone and pigtail tube drainage. The arrow in the lower panel I denoted lung consolidation and pleural effusion.

Computed tomography (CT) imaging revealed bilateral pleural effusion, bilateral interstitial pneumonia, multiple kidney stones, and severe right hydronephrosis (Fig. 1).

### 2.7. Laboratory examinations

Laboratory examinations showed low hemoglobin (Hb) levels (79 g/L), and high values of white blood cell count (22.56 × 10^9^ cells/L), B-type natriuretic peptide (1774 pg/mL), and C-reactive protein (93.60 mg/L).

## 3. Final diagnosis

The final diagnosis of the presented case was high-grade urothelial carcinoma.

## 4. Treatment

After the full assessment of her clinical conditions, the patient was initially admitted to the intensive care unit, where CT-guided nephrostomy tube placement was performed to drain the pus from the right kidney, and post-procedure carbapenem antibiotics were administered. After 6 days of medical treatments in the intensive care unit, the patient showed significant improvement and was transferred to a standard ward. There, the patient was treated with puncture drainage of the pleural effusion, blood and albumin transfusions as supportive treatments, antibiotics, and other methods for 21 days. Unfortunately, the treatments were unsatisfactory and the right renal abscess recurred and the UTI symptoms persisted. Additionally, systemic inflammatory response syndrome occurred. After a full clinical assessment, a surgical treatment involving a radical right nephrectomy/pyonephrosis-nephrectomy and ureteral stump resection was chosen. The patient and her family agreed to the treatment plan, and the surgical procedures were performed without the occurrence of a major procedure-related complication.

Postoperative pathological examination of the surgical specimen showed high-grade urothelial carcinoma, an overly aggressive variant of urothelial carcinomas. The tumor size was 10 × 7.5 × 8.5 cm with intratumoral necrosis and invasion of the renal parenchyma. Bleeding and degenerative necrosis were also observed under the microscope.

To prevent stress ulcer formation and bleeding after the surgery, postoperative care for the patient included enteral nutrition support therapy, proton pump inhibitors (PPIs), and other protective agents. On the first day post-surgery, the gastroscopy revealed an absence of abnormal GI findings (neither perforation nor bleeding).

**Figure 2.**
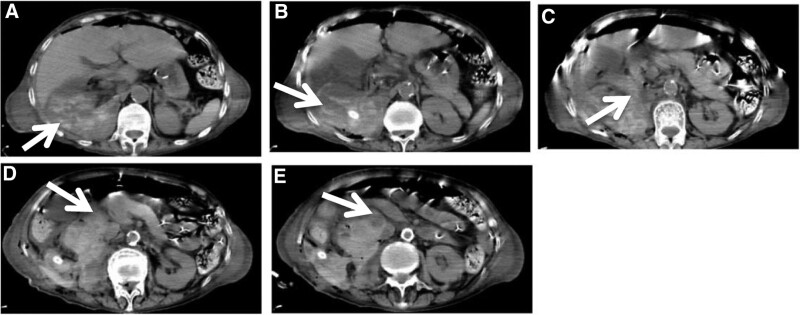
**Postoperative computed tomography (CT) images of retroperitoneal hematoma after radical nephrectomy and compression of the adjacent duodenum.** CT images showed the location and size of the retroperitoneal hematoma after radical nephrectomy. The retroperitoneal hematoma compressed the adjacent structures, including the duodenal wall. The arrows indicated the boundary of the hematoma (in all panels) and the relationship between the hematoma and the duodenum in panels C, D, and E.

Notably, on the second day after surgery, Hb levels were abnormally low (64 g/L), which could be attributable to the postoperative retroperitoneal hematoma of the surgical wound as revealed by a subsequent abdominal CT scan (Fig. 2). Meanwhile, there were no clinical signs of GI bleeding.

**Figure 3.**
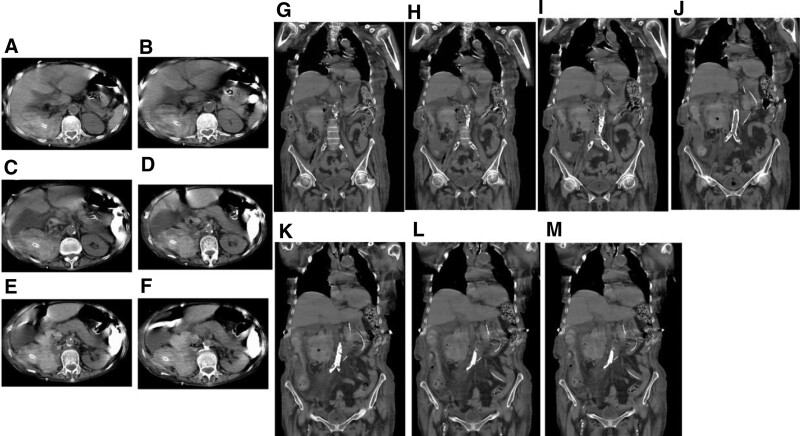
**Abdominal computed tomography (CT) images after radical right nephrectomy/pyonephrosis-nephrectomy and ureteral stump resection in the patient.** Abdominal CT imaging was performed following radical right nephrectomy and ureteral stump resection, and it revealed multiple instances of pneumoperitoneum and high-density shadows, suggesting a hematoma and gastrointestinal (GI) bleeding in the patient.

**Figure 4.**
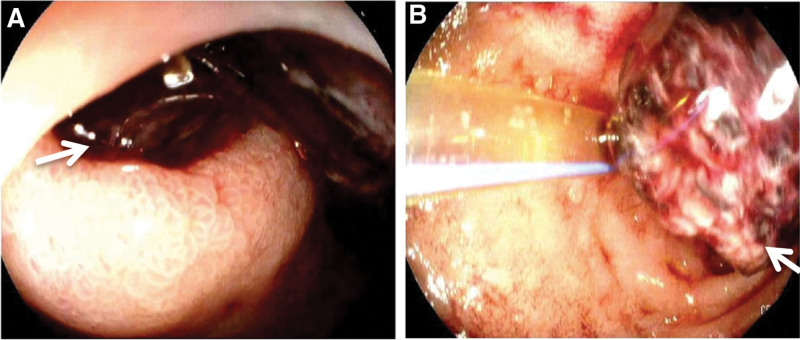
**Esophagogastroduodenoscopic images after radical right nephrectomy/pyonephrosis-nephrectomy and ureteral stump resection in the patient.** Esophagogastroduodenoscopy examinations of the lining of the upper gastrointestinal (GI) tract, including esophagus, stomach, and the bulbar and descending duodenum, showed normal mucosa of the esophagus and stomach, and ulceration, perforation, and bleeding of the duodenum. The arrows indicated the edge of the perforation (left panel) and the giant duodenal hematoma (right panel).

In the following days, Hb levels progressively decreased during the post-surgical hematoma absorption in the patient, meanwhile PPIs as part of the postoperative care remained in the prevention of ulcer formation. On day 14 post-surgery, a gastroscopic assessment was conducted, and the findings indicated duodenal ulcers in the bulbar and descending duodenum that nearly penetrated the muscularis mucosae and the submucosa to form a GI perforation. Abdominal CT imaging identified multiple instances of pneumoperitoneum and high-density shadows, suggesting a hematoma and GI bleeding in the patient (Fig. 3). The patient had other clinical signs of duodenal ulceration, perforation, and GI bleeding (Figs. 3 and 4). Esophagogastroduodenoscopy examinations of the lining of the upper GI tract, including the esophagus, stomach, and the bulbar and descending duodenum, revealed normal mucosa of the esophagus and stomach, and ulceration, perforation, and hemorrhaging of the duodenum (Fig. 4). Bloody and black stool were observed, suggesting massive GI bleeding in the patient.

## 5. Outcome and follow-up

Unfortunately, the patient died on day 15 after surgery.

This case study suggested that early initiation of preventive strategies, such as high-dose PPIs, may be essential to prevent this high-risk complication and the subsequent duodenal ulceration and life-threatening GI bleeding after a radical nephrectomy procedure. More importantly, once primary retroperitoneal hematoma is identified, timely treatment could be a critical strategy in the prevention of duodenal ulcers.

## 6. Discussion

This case study reported an unusual case involving the occurrence of a retroperitoneal hematoma and subsequent duodenal ulcerative bleeding in a 77-year-old woman who underwent a right radical nephrectomy for treatment of a severe recurrent renal abscess.

In this patient, a right renal abscess and UTI were initially considered as she underwent medical treatments, including drainage and antibiotics for the UTI commonly caused by a Gram-negative bacterial infection. The patient had underlying diseases, including hypertension, bilateral pleural effusion, bilateral interstitial pneumonia, kidney stones, UTI, etc, which, along with her advanced age, made the medical treatments less effective. In fact, all treatments appeared to be unsatisfactory, and the recurrent renal abscess remained in the patient. After a full clinical assessment and as agreed upon by the patient and her family, the surgical treatment consisting of a right radical nephrectomy/pyonephrosis-nephrectomy and ureteral stump resection was performed. It must be noted that urologic cancers were not detected in preoperative examinations at our hospital or previous hospitals. After a postoperative pathological examination was conducted on the surgical biopsy, high-grade urothelial carcinoma was diagnosed and confirmed in the patient.

**Figure 5.**
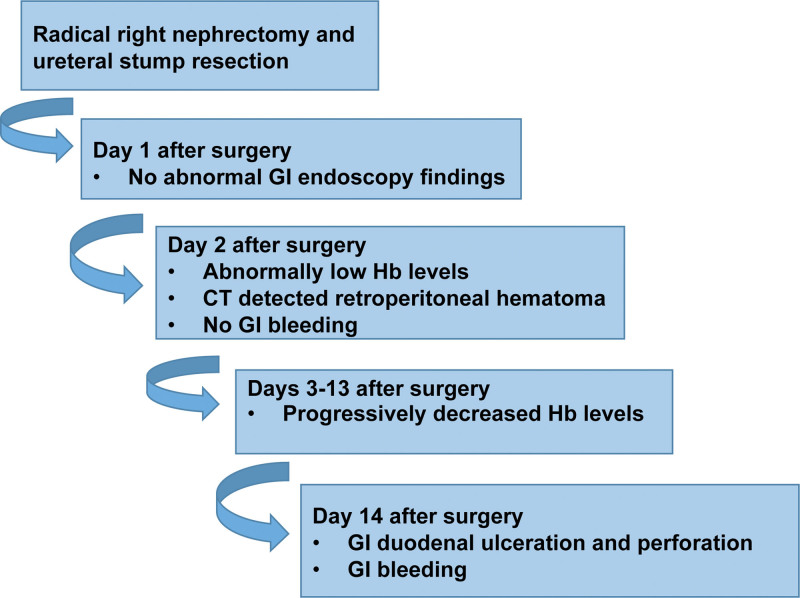
**Timeline of postoperative retroperitoneal hematoma and duodenal ulcerative bleeding.** CT = computed tomography, GI = gastrointestinal, Hb = hemoglobin.

Duodenal ulcers are commonly caused by *H pylori* infection. If left untreated, duodenal ulcers can progress into GI perforation and bleeding, manifesting as abdominal pain, and even present as bloody or black vomit or stool. Notably, duodenal ulceration and hemorrhaging occurred in our patient after the formation of a retroperitoneal hematoma, which occurred as a complication of the right radical nephrectomy for treatment of the severe renal abscess (Fig. 5). The duodenal ulceration and bleeding were indicated by the following findings in the patient: bloody and black stool were visible; Hb levels were abnormally low 2 days after surgery, and progressively decreased over the following days; multiple instances of pneumoperitoneum and high-density shadows on abdominal CT imaging suggested hematoma and GI bleeding; the site of GI bleeding was located in the bulbar and descending duodenum by gastroscopic examination and abdominal CT imaging; and duodenal ulceration and perforation that almost penetrated the intestinal wall was revealed under gastroscopy.

Given that GI bleeding can occur as a consequence of many GI disorders (e.g. gastric cancer) and the patient was postoperatively diagnosed to suffer from high-grade urothelial carcinoma, we ruled out the possibility of renal cell carcinoma/high-grade urothelial carcinoma metastasis to the duodenum/GI tract in pre- and post-operative abdominal CT imaging and GI endoscopic examinations. On abdominal CT 1 day after the surgery, a giant retroperitoneal hematoma was located just outside of the wall of the bulbar and descending duodenum. Meanwhile, a gastroscopy that was performed to guide nasogastric tube placement for early enteral feeding showed that the GI mucosa was in good condition. As part of the postoperative care, PPIs were used to prevent stress ulcer formation in this patient. As such, we postulated that the retroperitoneal hematoma might be related to the duodenal ulceration and massive bleeding in this patient. At present, it is important to note that the mechanisms underlying the association of a retroperitoneal hematoma with ulceration are unclear, but these could possibly involve chemicals or substances that were released during hematoma absorption. Previous studies have shown that eosinophilic peroxidase, eosinophilic cationic protein, and cytokines (e.g. interleukin-1, transforming growth factors) can induce tissue injury (e.g. ulcers) in the GI tract.^[3,4]^ Impaired fibrinolytic activity may also relate to the occurrence of peptic ulcers.^[5]^ In addition, there is a possibility that the fibrinolysis system was activated in the duodenum due to compression from the retroperitoneal hematoma, leading to the release of iron porphyrin and other relevant molecules from the hematoma and subsequently promoting duodenal ulceration and perforation in the patient. Wagner et al^[6]^ believe that a stroke is related to brain or subarachnoid hemorrhage and the release of large amounts of Hb outside the cell. Perhaps the patient duodenal ulcer and perforation were related to large amounts of Hb and iron porphyrin in very large peritoneal hematomas. It may merit attention in this case that the retroperitoneal hematoma might be associated with the duodenal ulceration and bleeding after the radial nephrectomy. The duodenal mucosa has a rich blood supply, and to maintain vascular patency, an active fibrinolytic system is necessary. Compression from a retroperitoneal hematoma may reduce blood flow into the duodenal mucosa, and therefore may lead to ischemia and ulceration of the duodenum. As such, the giant retroperitoneal hematoma following the operation could have induced the ulceration and perforation, and ulcerative hemorrhaging in the patient.

In this case, the patient developed a giant retroperitoneal hematoma after the radical nephrectomy procedure, which may have compressed the adjacent structures, such as the intestinal wall, resulting in the impairment of the fibrinolysis system and further promoting GI ulceration and perforation. O’Brien et al have suggested that compression of the GI tract wall may lead to the activation of the fibrinolytic system, subsequently releasing free plasmin, and local fibrinolysis may aggravate GI bleeding, while the increase of plasma fibrinolytic activity may cause abnormal hemorrhaging of blood vessels in the upper GI tract.^[7]^ Junquera et al indicated that a decrease of plasma plasminogen activator inhibitor type 1 is a risk factor for bleeding tendencies in patients with vascular hyperplasia.^[8]^

A retroperitoneal hematoma may compress the adjacent structures, in this case the duodenum, leading to ischemia. Ischemia is an uncommon cause of duodenal ulcers.^[9,10]^ Unlike a peptic ulcer caused by *H pylori* infection, the most common symptom in patients with GI ischemic ulcers is severe GI bleeding, and mild symptoms are rarely observed in ischemic ulcers with perforation.^[9]^ A further perception of such patients included resistance to therapy, and in combination with severe GI bleeding, these largely contribute to the lethal course, fatal bleeding, and poor clinical outcomes. Consistent with previous findings in patients with ischemic ulcers resistant to therapy, although interventions (including PPIs and gastric mucosal protective agents) were used to protect the gastric mucosa and prevent GI ulceration, the patient developed massive GI bleeding 14 days post-surgery. The severe GI bleeding in this case agreed with the common symptom observed in patients with ischemic GI ulcers. It has been noted that, without implementing any therapeutic interventions, the course of GI ischemic ulcers is varied, and can progress to fatal GI bleeding. It is still unclear if inhibitors of acid secretion could have beneficial effects in affected patients. Once the ulcer has formed, it would be an effective treatment to resect the involved duodenal segment.^[9,11,12]^

## 7. Conclusion

The experience and lesson in managing this patient should be added to previously reported aspects of radical nephrectomy as a surgical treatment with a retroperitoneal hematoma as a procedure-related complication. Early implementation of preventive strategies (e.g. high-dose PPIs) and prompt treatment of primary retroperitoneal hematoma could be critical in the prevention of duodenal ulcers in patients with similar conditions. Moreover, the findings in this case study suggested that fibrinolytic substances may be involved in duodenal ulceration.

## Author contributions

**Conceptualization:** Yong Luo, Zhigang Luo.

**Investigation:** Yong Luo.

**Resources:** Zhigang Luo.

**Supervision:** Zhigang Luo.

**Writing – original draft:** Yong Luo, Zhanchen Liao.

**Writing – review & editing:** Yong Luo, Zhanchen Liao, Qing Li, Zhigang Luo.
